# Mechanism of Action and Applications of Interleukin 24 in Immunotherapy

**DOI:** 10.3390/ijms17060869

**Published:** 2016-06-02

**Authors:** Leah Persaud, Dayenny De Jesus, Oliver Brannigan, Maria Richiez-Paredes, Jeannette Huaman, Giselle Alvarado, Linda Riker, Gissete Mendez, Jordan Dejoie, Moira Sauane

**Affiliations:** 1 Department of Biological Sciences, Herbert H. Lehman College, City University of New York, 250 Bedford Park Boulevard West, Bronx, NY 10468, USA; leah.persaud@lehman.cuny.edu (L.P.); dayenny.dejesus@lc.cuny.edu (D.D.J.); brannigan.oliver@gmail.com (O.B.); mariarichiez@gmail.com (M.R.-P.); galvarado7@gmail.com (G.A.); lindarr@optonline.net (L.R.); gissete.mendez@lc.cuny.edu (G.M.); jordan.dejoie@lc.cuny.edu (J.D.); 2 Department of Biology, The Graduate Center, City University of New York, 365 Fifth Avenue, Room 4315, New York, NY 10016, USA; jhuaman08@gmail.com; 3 Department of Biological Sciences, Hunter College, City University of New York, 695 Park Avenue (at 68th street) HN927, New York, NY 10065, USA

**Keywords:** IL-24, Sigma 1 Receptor, cancer, inflammatory disease, endoplasmic reticulum (ER) stress

## Abstract

Interleukin 24 (IL-24) is an important pleiotropic immunoregulatory cytokine, whose gene is located in human chromosome 1q32-33. IL-24’s signaling pathways have diverse biological functions related to cell differentiation, proliferation, development, apoptosis, and inflammation, placing it at the center of an active area of research. IL-24 is well known for its apoptotic effect in cancer cells while having no such effect on normal cells. IL-24 can also be secreted by both immune and non-immune cells. Downstream effects of IL-24, after binding to the IL-20 receptor, can occur dependently or independently of the JAK/STAT signal transduction pathway, which is classically involved in cytokine-mediated activities. After exogenous addition of IL-24, apoptosis is induced in tumor cells independently of the JAK/STAT pathway. We have shown that IL-24 binds to Sigma 1 Receptor and this event induces endoplasmic reticulum stress, calcium mobilization, reactive oxygen species generation, p38MAPK activity, and ceramide production. Here we review IL-24’s role in autoimmunity, infectious disease response, wound repair, and vascular disease. Detailed understanding of the pleiotropic roles of IL-24 signaling can assist in the selection of more accurate therapeutic approaches, as well as targeting of appropriate cell types in treatment strategy development, and ultimately achieve desired therapeutic effects.

## 1. Introduction

Interleukin 24 (IL-24) belongs to the IL-10 cytokine family, which consists of nine related molecules: IL-10, IL-19, IL-20, IL-22, IL-24, IL-26, IL-28A, IL-28B, and IL-29. These cytokines are classified into three subfamilies with different biological functions: The IL-10 subfamily represented by IL-10 itself; the IL-20 subfamily (IL-19, IL-20, IL-22, IL-24, and IL-26); and type III interferons (IFNs), IL-28A, IL-28B, and IL-29. IL-24 is released by both immune and non-immune cells [1]. It is produced by peripheral blood mononuclear cells (PBMC), mostly monocytes, and T and B cells. Non-immune cells that secrete IL-24 include cultured melanocytes, dermal keratinocytes, and IL-1 stimulated human colonic subepithelial myofibroblasts (SEMFs) [2].

In terms of its function, IL-24 possesses several major activities: It causes cancer-specific cell death when expressed at supra-physiologic levels; it exerts autoimmunity effects (psoriasis and other autoimmune disorders including rheumatoid arthritis, spondyloarthropathy, and inflammatory bowel disease); it suppresses keratinocyte proliferation during wound healing; it exerts a protective effect in cardiovascular disease and in bacterial infections. To carry out several of its functions, IL-24 can signal through two types of membrane receptors (IL-22R1/IL-20R2 and IL-20R1/IL-20R2) with concurrent activation of the Janus kinase (JAK)/signal transducer and activator of transcription (STAT) pathway within their cytoplasmic domains [3].

## 2. The Cellular Sources of IL-24 Expression

IL-24 is produced by immune cells, such as myeloid cells and lymphoid cells [4]. Based on *in vitro* experiments with established cell lines and primary cells cultures from patients, and animal studies, epithelial cells secrete IL-24 in response cytokine stimulation. Myeloid cells also produce IL-24 in response to microbial products, such as lipopolysaccharides (LPS), through the activation of Toll-like receptors (TLRs). In monocytes, the expression of IL-24 is induced by LPS, concanavalin A or cytokines [3]. To prevent uncontrolled T cell responses, it has been shown that IL-24 directly acts on T cells to possibly dampen the first rounds of CD8 cell expansion to avoid uncontrolled T cell responses [5]. Additionally, B lymphocytes induce the expression of IL-24 after combined anti-IgM and CD40-L stimulation [6]. In response to cytokines secreted by immune cells, non-lymphoid cells can also produce IL-24. Melanocytes produce physiological levels of IL-24, whereby its expression progressively decreases during the transformation of melanocytic cells to metastatic melanoma [7]. IL-1β stimulation also increases IL-24 expression in keratinocytes and human colonic SEMFs [2]. Table 1 summarizes the celluar sources and targets of IL-24.

## 3. IL-24 and Its Receptors

**Figure 1:**
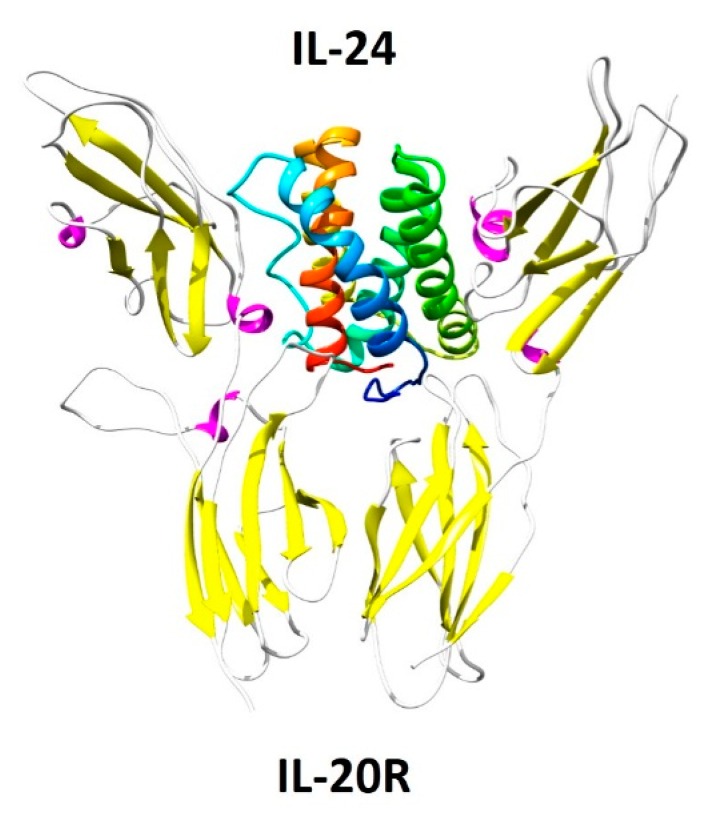
Three-dimensional structure of hIL-24 with its IL-20R receptor. Using the known crystal structures of IL-19, IL-22, and IL-20, a stable three-dimensional structure of human IL-24 (hIL-24) was obtained using computer modeling. When bound to each other, IL-20R receptor chain (yellow and pink) and hIL-24 share eight hydrogen bonds and 102 non-bonded interactions similar to IL-20 and IL-20R receptor chain [74].

Numerous investigations, including expression profiling in patients’ cells and *in vitro* and animal experiments, suggest that IL-24’s functions of which includes antibacterial responses, tissue remodeling, wound healing, and anti-tumor effects, require different heterodimeric receptors. IL-24 signals through the IL-20 receptor β-subunit (IL-20RB). IL-20RB can form a functional heterodimeric receptor with either the IL-20 receptor α-subunit (IL-20RA), or with the α1 subunit of the IL-22 receptor (IL-22RA1) [8]. Figure 1 illustrates the three-dimensional structure of IL-24 and its IL-20 receptor. Signaling induced by IL-24 binding to the IL-20 receptor complex is known to occur primarily via the JAK pathway and subsequent activation of STAT 1 and 3 signaling pathways [9]. Additionally, IL-24 activates extracellular signal-regulated kinases (ERKs), JUN N-terminal kinase (JNK) and p38 mitogen-activated protein kinase (p38MAPK) pathways [10]. The expression of IL-20RA and IL-22RA1 receptors is limited to certain tissues and they are mostly absent from hematopoietic lineages. IL-22RA1 is highly expressed in the skin, pancreas, liver, kidneys and intestine (Reviewed in [11]). IL-20RA and IL-20RB are present in the skin, lungs, ovary, testes and placenta, but their expression is low in the intestine and liver (Reviewed in [12]). In addition to these functional receptors, the Sigma 1 Receptor (Sig1R), is overexpressed in cancer cells [13]. IL-24 binds to Sig1R, and this initial binding is key in IL-24-cancer-specific apoptosis, as we have shown [14]. Interestingly, Sig1R is a unique protein that can modulate many biological mechanisms associated with inflammation and cancer-specific apoptosis.

In accordance with what has been observed with IL-24, the combination of immunosuppression and anti-inflammatory properties makes Sig1R ligands attractive molecules for therapeutic applications like in autoimmune diseases where both immune and inflammatory processes are involved. Interestingly, Sig1R is known to translocate and remodel the plasma membrane [15]. Accumulating evidence indicates that Sig1R is overexpressed in many cancer cell lines, and contributes to the invasion and metastasis in many human tumors. Aydar *et al.* demonstrated that Sig1R is associated with β_1_ integrin in lipid cholesterol-enriched rafts in breast cancer cells [16]. In response to environmental conditions encountered in cancer tissue, Sig1R may dynamically trigger various adaptation mechanisms, which is tightly dependent on the client protein available in a given tumor cell type. The discovery of surface localization of BiP and Sig1R in cancer cells reveals potential novel functions, interactions with cell-surface receptors, and possible therapeutic implications [17]. Therefore, it is tempting to speculate that IL-24 protein binds to Sig1R in the plasma membrane, a possibility we are currently addressing. We have demonstrated that Sig1R is the upstream initial signal transduction molecule common to cascades of events involving IL-24-induced endoplasmic reticulum stress (ER-stress) dependent and independent downstream pathways [14]. In summary, the identification of Sig1R as a key initial mediator of IL-24-cancer-specific apoptosis significantly broadens the therapeutic potential of Sig1R and IL-24 for tumors and provides important knowledge for our understanding of IL-24 as an immune modulating cytokine.

## 4. Interleukin-24 in Cancer

IL-24 is an immunomodulatory cytokine which can also display broad cancer-specific suppressor effects. The tumor suppressor activities of IL-24 include inhibition of angiogenesis, sensitization to chemotherapy, and induction of cancer-specific apoptosis. Given its ubiquitous apoptotic effect on malignant cells, lack of an effect on normal cells, and absence of significant side effects, IL-24 is an important candidate for cancer therapy. Early-phase I human studies have shown encouraging results [18,19]. Our previous studies have shown that a non-replicating adenoviral vector expressing IL-24 (Ad.IL-24) induces apoptosis selectively in cancer cells through IL-20 receptor-dependent, JAK/STAT-independent and MAPK-dependent pathways [9]. We have demonstrated that IL-24 is able to induce apoptosis via both intracellular and extracellular signaling mechanisms [20]. In addition, we have shown that Ad.IL-24 and IL-24 protein (generated from Ad.IL-24-infected cells) display a broad cancer-specific pro-apoptotic activity through induction of ER stress, and ceramide production [21]. Moreover, we have established that production of ceramide and induction of ER stress following Ad.IL-24 infection or IL-24 treatment occurs specifically in cancer but not in normal cells. In addition, secreted IL-24 protein induces a robust expression of endogenous IL-24 and subsequent induction of tumor-specific killing through an ER stress-mediated pathway as well as by ROS production [22]. Specifically, IL-24 protein induces stabilization of its own mRNA without activating its promoter. We have also shown that Ad.IL-24 induces p38MAPK [23]. Consistent with these reports, Otkjaer and colleagues have recently shown that p38MAPK regulates IL-24 expression by stabilization of the 3′UTR of their mRNA [24]. In recent years, a consensus has established that ER stress is the initial pathway in IL-24-induced apoptosis.

The IL-24 gene can also be alternatively spliced producing different isoforms of IL-24. FISP, the IL-24 murine homolog in T helper 2 lymphocytes, is also alternatively spliced. The FISP-sp isoform, which has 29 nucleotides deleted from the 5′-end of exon 4, has been shown to dimerize with full length IL-24 to antagonize its apoptotic activity *in vivo* [25]. In a human osteosarcoma U2OS cell line, Whitaker *et al.* were able to identify and characterize five alternatively spliced variants [26]. One of these variants, mda-7/IL-24δ2,3,5, induced higher levels of apoptosis in the U2OS cancer cells compared to full-length IL-24 as evidenced by caspase 3 and 7 activation. Mda-7/IL-24δ2,3,5 lacks exons 2, 3, and 5 and has been shown to be favorably expressed over full-length IL-24 when splice factor SRp55 protein levels are increased [27].

Secreted IL-24 protein, generated from Ad.IL-24-infected cells, promotes anti-angiogenic, immunostimulatory, radiosensitizing and “bystander” antitumor activities. IL-24 stimulates the immune system to generate secondary cytokines, such as TNF-α, IFN-c, and IL-1, which evoke an antitumor immune response [28]. Secreted IL-24 protein, generated from Ad.IL-24-infected cells, exerts anti-angiogenic activity by inhibiting endothelial cell differentiation and by blocking the activities of VEGF and TGF-α via inhibition of Src activity within tumor cells [29]. We have demonstrated that IL-24 protein generates additional molecules of IL-24, inducing more ER-stress and culminating in an untenable imbalance resulting in apoptosis in cancer cells [22]. Specifically, exogenous IL-24 protein induces growth inhibition and apoptosis only in cancer cells through a mechanism identical to Ad.IL-24 infection. These findings have important implications for our understanding of IL-24 as a tumor suppressor protein as well as an immune modulating cytokine.

More recently, we have shown that Sigma 1 Receptor (Sig1R) interacts with IL-24 and that this IL-24:Sig1R interaction mediates, and is critical, for apoptosis induction by IL-24 [14]. These studies defined Sig1R as a key initial mediator of IL-24 induction of cancer-specific cell killing and have important implications for understanding IL-24 as a tumor suppressor protein, as well as an immune modulating cytokine. In addition to virus-administered IL-24, recombinant IL-24 protein induces apoptosis, inhibition of angiogenesis, sensitization to chemotherapy, and immunotherapeutic properties. Phase I trials of intratumoral injections of Ad.IL-24 (INGN241) have demonstrated that Ad.IL-24 is well tolerated and induces apoptosis in tumor cells with s significant clinical activity [18]. Similar results were seen in a dose-escalation study of Ad.IL-24 where tumor cells underwent apoptosis in patients after successful gene transfer of IL-24. [19]. The transition of IL-24 into a phase II clinical trial reinforces the hypothesis that IL-24 is safe and affords remarkable potential as a cancer gene therapeutic agent [30].

Oncolytic adenoviruses (OAs) expressing IL-24 have also been used to treat a variety of human cancer cell lines with no effect on normal cells. OAs replicate selectively in tumor cells and function to direct the host’s innate and adaptive immune responses to the tumor (Reviewed in [31]). Potent antitumor activity of OAs expressing IL-24 has been described in colorectal, hepatocellular, nasopharyngeal, lung, and cervical carcinomas, melanoma, and in a pancreatic cancer model in mice [32,33,34,35,36,37,38]. Several studies have also reported increased sensitivity of human liver, lung, colorectal, cervical, nasopharyngeal and osteosarcoma cancer cells to therapeutic drugs when treated with OAs expressing IL-24 [39,40,41]. These drugs include cisplatin, dichloroacetate, temozolomide, and doxorubicin. In a mouse model, oncolytic adenovirus expressing both miRNA-34a and the IL-24 gene was shown to induce antitumor activity in xenograft hepatoceullar carcinoma tumors [42]. While these results are promising, further *in vivo* studies examining the efficacy of OAs expressing IL-24 are needed.

An important question, which remained unresolved, is why IL-24 has the abilities to selectively induce apoptosis in a large spectrum of human cancer-derived cell lines without harming normal cells. One possible reason for this differential killing effect involves inherent biochemical differences between normal and cancer cells (ER stress, ROS production and ceramide), another possibility is that IL-24 is able to target a molecule that only triggers apoptosis in cancer cells. The third option for this differential killing effect is that both of the above hypotheses are correct. The molecular properties of IL-24 and associated pathways will need to be analyzed in much greater detail to fully understand this cytokine. We therefore searched for a candidate molecule that can consolidate the entire signal transduction pathway triggered by IL-24. In particular, it is postulated that IL-24 not only triggers ER stress and its downstream molecules (ROS, ceramide, calcium mobilization) but that IL-24 can also regulate gene products involved in invasion (β-catenin, FAK, and PI3K/PKB), angiogenesis (VEGF and TGF-β), and metastasis (β-integrin). Notably, IL-24 activates TRAIL and Fas-FasL (Reviewed in [43,44]). This is remarkably similar to the effects reported for Sigma 1 Receptor antagonists [45]. Furthermore, recent studies identified that Sigma 1 Receptor binds to BiP [17] and regulates ER stress, calcium mobilization, and ROS production [14]. Our studies define Sigma 1 Receptor as a key initial mediator of IL-24 induction of cancer- specific killing.

## 5. Anti-Angiogenic Properties of IL-24

Ramesh *et al.* demonstrated for the first time that IL-24-mediates anti-angiogenic activity in lung tumor xenograft by reducing the number of CD31 positive endothelial cells, a marker indicative of reduced blood vessels in the tumor [29]. Similarly, it has been shown that addition of purified human IL-24 protein to human umbilical vein endothelial cells (HUVEC) and human lung microvascular endothelial cells (HMVEC-L) exerts an inhibitory effect on endothelial cell differentiation (ECD) but not endothelial cell proliferation. *In vitro* studies clearly reveal that IL-24 induces an inhibitory effect on endothelial cell differentiation in human umbilical vein endothelial cells (HUVEC) through a IL-22R-dependent and Akt/mTOR-dependent pathways.

In addition to reduced number of CD31 positive endothelial cells, inhibition on endothelial cell differentiation IL-24 can also modulate angiogenesis by suppressing growth factors, such as vascular endothelial growth factor (VEGF), IL-8, fibroblast growth factor (FGF) and transforming growth factor (TGF), produced by tumor cells [29]. These findings demonstrated that IL-24 protein directly possess anti-angiogenic activity.

## 6. Metastasis

IL-24 inhibits the production of matrix metalloproteinase (MMP)-2, -9, and focal adhesion (FAK) protein expression, essential mediators for metastasis progression [46]. Besides these suppressive activities, IL-24 favors E-Cadherin expression [47]. E-Cadherin represents a family of membrane receptors that mediate calcium-dependent homophilic cell-to-cell adhesion. In addition, the inhibitory activity of IL-24 on cell migration and invasion is mediated via inhibition of PI3K-AKT-mTOR and Wnt signaling pathways [48,49]. IL-24 can also inhibit the Akt/mTOR pathway by inhibiting the CXCR4/CXCL12 pathway [50,51]. Overall, these studies show that IL-24 is an important anti-metastatic molecule.

## 7. IL-24 and Inflammatory Diseases

Various lines of evidence indicate that IL-24 plays a role in immune-pathological diseases, including psoriasis, rheumatoid arthritis, and inflammatory bowel disease (IBD).

### 7.1. Psoriasis

Psoriasis is a chronic inflammatory skin condition resulting from a complex interplay among the immune system, keratinocytes, susceptibility genes, and environmental factors. The presence of IL-24 along with IL-19 and IL-20 has been observed in psoriatic skin lesions via *in situ* hybridization assays where increased expression of IL-24 has been detected in psoriatic skin compared to normal skin [52]. IL-24 induces several psoriasis-associated factors that promote inflammation and epidermal hyperplasia, such as chemokines, pro-inflammatory cytokines, S100 family proteins, β-defensins and proteins involved in tissue remodeling, including marapsin (also known as PRSS27) and the kallikreins [53].

It has been demonstrated that overexpression of IL-24 in transgenic mice lead to the development of skin lesions similar to those seen in human psoriasis [52]. IL-24 transgenic mice exhibited infiltrating macrophages in the dermis with concomitant increases in monocyte chemoattractant protein-1 (MCP-1), a key chemokine that regulates migration and infiltration of monocytes/macrophages from both keratinocytes in the epidermis and immune infiltrates in the adjacent dermal layer below. It has been suggested that NF-κB plays a role in inflammatory processes through the observation that epidermis-specific inhibition of NF-κB activates STAT3 and increases IL-24 expression in TNF-stimulated human primary keratinocytes. It was demonstrated that skin treated with anti-TNF exhibits a downregulation of IL-24 expression in psoriasis patients, making the epidermal keratinocyte a direct target of pathogenic TNF signaling in psoriasis. Altogether, the studies of psoriasis and IL-24 support the idea that IL-24 plays a significant role in the expression of pro-inflammatory mediators resulting in psoriatic skin lesions thus, providing evidence that IL-24 is a key factor in the initiation of psoriasis.

### 7.2. Rheumatoid Arthritis

Rheumatoid arthritis (RA) is a chronic autoimmune disorder that primarily affects joints. RA typically manifests with signs of inflammation, with the affected joints being swollen, warm, painful and stiff. Kragstrup *et al.* first demonstrated the association between IL-24 expression and rheumatic diseases, specifically RA and spondyloarthropathy [54]. Spondyloarthropathy (SpA) is a group of related diseases that exhibit spinal inflammation and peripheral joint oligoarthritis. The related diseases include psoriatic arthritis, reactive arthritis, and entereopathic arthritis associated with inflammatory bowel disease. It was found that IL-24 protein was 1.5- to 2.5-fold higher in the synovial fluid samples from patients with RA and SpA in comparison to samples from healthy control and non-inflammatory disease patients. The authors also studied the levels of IL-20 in the synovial fluid samples since both IL-20 and IL-24 share receptor complexes, IL-20R1/IL-20R2 and IL-22R/IL-20R2. When compared to IL-20, the level of IL-24 was approximately 10-fold higher in the synovial fluid of RA and SpA patients indicating that IL-24 may be playing a more active role in the joint. Immunohistochemistry analysis demonstrated that IL-24 was detectable in the endothelial cells of the synovial blood vessels and mononuclear cells of all synovial membranes from RA patients. IL-24 and IL-20 was found to induce the production of MCP-1 in synovial fluid mononuclear cell cultures. Furthermore, a significant positive correlation was found between IL-24 and IL-20 concentrations and MCP-1 concentration in synovial fluid and plasma samples of both RA and SpA patients. Clearly, IL-24 is associated with RA diseases but more research is required to fully understand the biology of IL-24 in rheumatoid arthritis.

### 7.3. Inflammatory Bowel Disease

Inflammatory Bowel Disease (IBD) is a chronic, relapsing inflammatory disorder of the gastrointestinal tract, which mainly includes ulcerative colitis and Crohn’s disease. It is thought that IBD is triggered by abnormalities in genetic and environmental factors. Andoh *et al.* demonstrated expression of IL-24 in inflamed mucosa of IBD patients and showed that IL-24 stimulates MUC gene expression via JAK1/STAT3 activation, contributing to a protective role in the mucosa from IBD patients [2]. In particular, colonic epithelial cells are targets of IL-24 in the mucosa and IL-24 plays anti-inflammatory and protective roles in intestinal mucosa. More recently, a clinical study demonstrated expression of IL-19 and IL-24 at the gene and protein expression levels in tissue and peripheral cells of patients exhibiting active or inactive Crohn’s Disease (CD) or active or inactive ulcerative colitis (UC) [55]. The study found that IL-19 and IL-24 gene expression was upregulated in patients with active IBD *versus* the inactive disease and non-inflammatory control groups. In addition, cells producing IL-24 and IL-19 were increased in active Crohn’s Disease patients in comparison to control patients and those with active ulcerative colitis. IL-24 was synthesized by peripheral B cells, CD4+ T cells, CD8+ T cells and monocytes in patients with active disease. Overall, IL-24 can promote a suppressive inflammatory effect on colonic epithelial cells and mucosal inflammation in IBD.

## 8. IL-24 in Host Defense

IL-24 induces innate defense mechanisms in epithelial tissues, during infection and inflammation, and restores tissue homeostasis. This function is mediated by promoting the production of various antimicrobial peptides including S100 family proteins, the calprotectin subunits S100A8 and S100A9 [56]. By inducing cytokine and chemokine production in epithelial cells, IL-24 facilitates the recruitment and activation of leukocytes at the site of inflammation [57].

IL-24 also plays a role in host defense during bacterial infections. IL-24 has been shown to have a protective effect against *Salmonella typhimurium* infection in a mouse model of typhoid fever [58]. In this role, IL-24 activates CD8+ T cells by stimulating neutrophil production of IFN-γ and IL-12 cytokines and nitric oxide leading to host resistance against Salmonella infection *in vivo*. In response to *Pseudomonas aeruginosa* infection, tracheal gland cells, which may play a key role in the pathogenesis of cystic fibrosis, strongly upregulate IL-24 expression among other IFN-γ associated genes [59]. The relationship between IFN-γ expression and IL-24 has also been reported in patients infected with *Mycobacterium tuberculosis* [60]. In comparison to individuals with latent tuberculosis infection, peripheral blood mononuclear cells (PMBCs) from tuberculosis patients had lower levels of IL-24 and IFN-γ. Exogenous addition of IL-24 to (PMBCs) from tuberculosis patients increased the expression of IL-12 family cytokines, including IL-12α, IL-12β, IL-23α and IL-27 leading to the increase of IFN-γ expression indicating that IL-24 plays a role in the immune response following infection with *M. tuberculosis*.

In addition to playing a protective role in the host defense in epithelial tissues during infection, IL-24 is activated during *Staphylococci* infections of the skin [61]. In particular, when *S. aureus* infects the skin, the host-defense response is dependent on the early production by skin cells of IL-1β and IL-17 (Reviewed in [62,63]), leading to neutrophil recruitment. Interestingly, IL-24 is induced locally by *S. aureus*, inhibits IL-1β and IL-17 production, leading to more severe infection and reduced neutrophil recruitment [64]. This gives IL-24 a dual-role in host defense, where it can be both protective and antagonistic depending on the type of bacterial infection.

## 9. Wound Repair

IL-24 has been found to be in wounds in migrating keratinocytes but not in the proliferating cells at the dermal-epidermal junction [65]. Normal healthy human skin samples do not exhibit IL-24 expression in keratinocytes. The expression of IL-1β, an important mediator of the inflammatory response, has been shown to increase the expression of IL-24 by 10-fold in keratinocytes. Poindexter *et al.* observed that IL-24 plays a role in preventing the proliferation and migration of keratinocytes by inhibiting TGFα [66]. Wounded human biopsy samples immunostained for IL-24 displayed positive staining in macrophages and keratinocytes. Several cytokines and growth factors, TGFα, TGFβ, IFNβ and IFNγ stimulated the secretion of IL-24 in natural human epidermal keratinocytes (NHEKs). *In vitro* assays also determined that when TGFα was added to wounded NHEKs cells, IL-24 interfered with the ligand’s rapid wound closure activity and prevented migration of the keratinocytes. Inhibitors of IL-24 and its receptor subunits, IL-20R1 and IL-22R1, were also used to confirm that IL-24 was inhibiting the activity of TGFα. Inhibiting IL-20R1 allowed wound closure in the presence of TGFα. Thus, IL-24 signals through IL-20R1/IL-20R2 to inhibit migration of keratinocytes. Migration and proliferation assays further showed that IL-24 blocks the proliferation and migration of TGFα treated NHEKs. A more recent study has shown that in human acute and chronic wound tissues, IL-24 significantly slows the migration of keratinocytes and this inhibition is mediated through an AKT-dependent pathway [65]. On the contrary, IL-24 has been shown to promote migration in human vascular endothelial cells (HECV) [67]. The authors of this study suggest that the pro-migratory effect may be related to AKT signaling because they found that treatment with a small-molecule AKT inhibitor could partially reduce the pro-migratory effects. Further research would need to be done to confirm this effect but overall it seems that IL-24 contributes to both pro- and anti-migratory effects depending on tissue or cell type, or the presence of growth factors. Recombinant IL-24 has also been demonstrated to induce pro-migratory effects of monocytes and neutrophils *in vitro* [4]. In the presence of pertussis toxin, migration was reduced suggesting that G-coupled receptors may also be involved in the pro-migratory activity of IL-24.

Overall, although elevated levels of IL-24 and its receptor are induced in wounded skin in both mice and humans, it is not completely clear the role IL-24 plays in wound healing. IL-24 accelerates inflammation by stimulating keratinocytes to express chemokines and cytokines that recruit macrophages and other leukocytes. Additionally, IL-24 promotes re-epithelialization process by acting on epidermal keratinocytes through the induction of keratinocyte growth factor (KGR, also known as FGF7). Although those responses favor the hypothesis that IL-24 is beneficial for wound closure, on the other hand, IL-24 appears to promote wound chronicity via its inhibitory effect on the migratory behavior of human keratinocytes, mediated through an AKT-dependent pathway. It is temping to speculate that uncontrolled tissue repair processes triggered by IL-24 cause this latter effect.

## 10. Cardiovascular Disease

One hallmark of cardiovascular disease is vascular calcification, which is the regulated process of biomineralization of vascular smooth muscle cells (VSMCs) similar to bone mineralization in osteogenesis. It is a clinical indicator of atherosclerosis and correlates greatly with cardiovascular disease mortality. Lee *et al.* performed a study to examine the role of IL-24 in cardiovascular disease, specifically VMSC calcification [68]. In a β-glycerophosphate (β-GP)-induced rat VSMC calcification model, recombinant human IL-24 was shown to inhibit β-GP-induced VSMC calcification. The authors showed that IL-24 suppressed β-GP-induced apoptosis of VSMCs and inhibited the expression of calcification and osteoblast markers by downregulating Bone morphogenic protein- 2 (BMP-2) and the Wnt/β-catenin pathway, which is important in the pathogenesis of both atherosclerosis and cancer. These results indicate a novel protective role of IL-24 in cardiovascular disease.

In another study, genetically engineered adenovirus modified to deliver IL-24 (Ad.IL-24) was also found to selectively suppress the growth and migration of rat pulmonary arterial smooth muscle (PAC1) cells *in vitro* [69]. In an atypical PAC1 cell line containing chromosomal aberrations treated with Ad.IL-24, both early and late apoptotic activity was detected. These results suggest that IL-24 could be used as a treatment for vascular proliferative disorders. Lee *et al.* later showed that exogenous administration of IL-24 attenuated the expression of vascular inflammation and hypertension-related genes in mouse vascular smooth muscle (MOVAS) cells [70]. Like vascular calcification, hypertension is also a hallmark of cardiovascular disease. In spontaneously hypertensive rats, 16 differentially regulated genes were identified including IL-24, which were previously not implicated in cardiovascular disease. IL-24 also regulates the expression of inflammation- and hypertension-related genes, such as angiotensinogen, endothelin-1, ATRAP and PDGF in H_2_O_2_ treated MOVAS cells. Overall, these studies suggest that IL-24 may be a novel therapeutic target for cardiovascular disease and/or hypertension.

One recent study has suggested that IL-24 polymorphisms are associated with metabolic and cardiovascular risk factors [71]. The study analyzed genetic factors of premature coronary artery disease (CAD) patients and those with subclinical atherosclerosis (SA). All participants were of Mexican Mestizo descent. The findings revealed an association of four polymorphisms of IL-24 (rs1150253, rs1150256, rs1150258, rs3762344) with cardiovascular risk factors in all categories of participants. Specifically, rs1150253 and rs1150258 polymorphisms produce DNA binding sites for some transcriptional factors suggesting that these IL-24 polymorphisms are associated with cardiovascular risk factors in the studied population.

Based on the current literature, it seems that IL-24 has a diverse role in cardiovascular disease. IL-24 has been shown to suppress calcification and osteoblast marker expression to promote the growth of vascular smooth muscle cells, which associates it to the pathogenesis of atherosclerosis. Conversely, IL-24 has been shown to selectivity inhibit rat pulmonary arterial smooth muscle cells indicating a possible role for it in the treatment of vascular disorders. In addition, IL-24 polymorphisms have been linked to cardiovascular and metabolic risk factors supporting the relationship between IL-24 and inflammatory diseases.

## 11. Discussion

IL-24 is a pleiotropic cytokine with effects on numerous cell populations including immune cells, epithelial cells, and cancer cells. IL-24 displays a broad range of activities including antibacterial responses, tissue remodeling, wound healing, and anti-tumor effects. The effects of IL-24 seem to be quite complex because its role can vary depending on the cellular source, target, and phase of the immune response. Overall, we can consider IL-24 as an immunoregulatory cytokine and as an antitumor molecule with a broad range of activities including, cancer-specific induction of apoptosis and inhibition of tumor angiogenesis. IL-24 actions rely on the binding to its molecular partners including BiP/GRP78 [72], Sig1R [14], IL-20 Receptors [3], and dsRNA-dependent protein kinase (PKR) [73]. It remains unclear whether the antitumor effects of IL-24 reflect its other biological functions. The molecular properties of IL-24 and associated pathways will need to be analyzed in much greater detail to fully understand this cytokine.

Overall, it can be seen that both the endoplasmic reticulum stress pathway and inflammation has an important role in IL-24 functions. ER stress and inflammation are related in a way that, under acute triggers, they function to safeguard cellular viability. However, when chronically induced, ER stress and inflammation are destructive and go beyond physiological control. ER stress-induced inflammation primarily serves to limit the tissue damage and facilitate tissue repair, however, it largely depends on the target cell type, the disease stage, and the type of ER stressor. It is anticipated that further clarification of the molecular events associated with IL-24 will enable novel avenues for therapeutic intervention in both cancer and inflammatory diseases.

## Figures and Tables

**Table 1:** Cellular sources and targets of IL-24.

Inducers of Expression	Cellular Sources	Transcription Factors	Receptor
IL-1β	Monocytes	Jak1	IL-20R1/IL-20R2
IL-17	Melanocytes	Tyrosine kinase 2	IL-22R1/IL-20R2
IL-22	TH2 lymphocytes	Stat 1	-
LPS	Fibroblasts Keratinocytes	Stat 3	-

## References

[1] Hofmann S.R., Rösen-Wolff A., Tsokos G.C., Hedrich C.M. (2012). Biological properties and regulation of IL-10 related cytokines and their contribution to autoimmune disease and tissue injury. Clin. Immunol..

[2] Andoh A., Shioya M., Nishida A., Bamba S., Tsujikawa T., Kim-Mitsuyama S., Fujiyama Y. (2009). Expression of IL-24, an activator of the JAK1/STAT3/SOCS3 cascade, is enhanced in inflammatory bowel disease. J. Immunol..

[3] Wang M., Tan Z., Zhang R., Kotenko S.V., Liang P. (2002). Interleukin 24 (MDA-7/MOB-5) signals through two heterodimeric receptors, IL-22R1/IL-20R2 and IL-20R1/IL-20R2. J. Biol. Chem..

[4] Buzas K., Oppenheim J.J., Zack Howard O.M. (2011). Myeloid cells migrate in response to IL-24. Cytokine.

[5] Wahl C., Müller W., Leithäuser F., Adler G., Oswald F., Reimann J., Schirmbeck R., Seier A., Weiss J.M., Prochnow B. (2009). IL-20 receptor 2 signaling downregulates antigen-specific T cell responses. J. Immunol..

[6] Maarof G., Bouchet-Delbos L., Gary-Gouy H., Durand-Gasselin I., Krzysiek R., Dalloul A. (2010). Interleukin-24 inhibits the plasma cell differentiation program in human germinal center B cells. Blood.

[7] Jiang H., Lin J.J., Su Z.Z., Goldstein N.I., Fisher P.B. (1995). Subtraction hybridization identifies a novel melanoma differentiation associated gene, *mda-7*, modulated during human melanoma differentiation, growth and progression. Oncogene.

[8] Parrish-Novak J., Xu W., Brender T., Yao L., Jones C., West J., Brandt C., Jelinek L., Madden K., McKernan P.A. (2002). Interleukins 19, 20, and 24 signal through two distinct receptor complexes. Differences in receptor-ligand interactions mediate unique biological functions. J. Biol. Chem..

[9] Sauane M., Gopalkrishnan R.V., Lebedeva I., Mei M.X., Sarkar D., Su Z.Z., Kang D.C., Dent P., Pestka S., Fisher P.B. (2003). Mda-7/IL-24 induces apoptosis of diverse cancer cell lines through JAK/STAT-independent pathways. J. Cell. Physiol..

[10] Tian H., Zhang D., Gao Z., Li H., Zhang B., Zhang Q., Li L., Cheng Q., Pei D., Zheng J. (2014). MDA-7/IL-24 inhibits Nrf2-mediated antioxidant response through activation of p38 pathway and inhibition of ERK pathway involved in cancer cell apoptosis. Cancer Gene Ther..

[11] Dudakov J.A., Hanash A.M., van den Brink M.R. (2015). Interleukin-22: Immunobiology and pathology. Annu. Rev. Immunol..

[12] Sabat R., Wallace E., Endesfelder S., Wolk K. (2007). IL-19 and IL-20: Two novel cytokines with importance in inflammatory diseases. Exp. Opin. Ther. Targets.

[13] Xu D., Yi W., Chen Y., Ma L., Wang J., Yu G. (2014). Overexpression of Sig1R is closely associated with tumor progression and poor outcome in patients with hilar cholangiocarcinoma. Med. Oncol..

[14] Do W., Herrera C., Mighty J., Shumskaya M., Redenti S.M., Sauane M. (2013). Sigma 1 Receptor plays a prominent role in IL-24-induced cancer-specific apoptosis. Biochem. Biophys. Res. Commun..

[15] Su T.P., Su T.C., Nakamura Y., Tsai S.Y. (2016). The Sigma-1 receptor as a pluripotent modulator in living systems. Trends Pharmacol. Sci..

[16] Palmer C.P., Mahen R., Schnell E., Djamgoz M.B., Aydar E. (2007). Sigma-1 receptors bind cholesterol and remodel lipid rafts in breast cancer cell lines. Cancer Res..

[17] Ortega-Roldan J.L., Ossa F., Schnell J.R. (2013). Characterization of the human sigma-1 receptor chaperone domain structure and binding immunoglobulin protein (BiP) interactions. J. Biol. Chem..

[18] Cunningham C.C., Chada S., Merritt J.A., Tong A., Senzer N., Zhang Y., Mhashilkar A., Parker K., Vukelja S., Richards D. (2005). Clinical and local biological effects of an intratumoral injection of mda-7 (IL24; INGN 241) in patients with advanced carcinoma: A phase I study. Mol. Ther..

[19] Tong A.W., Nemunaitis J., Su D., Zhang Y., Cunningham C., Senzer N., Netto G., Rich D., Mhashilkar A., Parker K. (2005). Intratumoral injection of INGN 241, a nonreplicating adenovector expressing the melanoma-differentiation associated gene-7 (mda-7/IL24): Biologic outcome in advanced cancer patients. Mol. Ther..

[20] Sauane M., Lebedeva I., Su Z., Choo H., Randolph A., Valerie K., Dent P., Gopalkrishnan R.V., Fisher P.B. (2004). Melanoma differentiation associated gene-7/interleukin-24 promotes tumor cell-specific apoptosis through both secretory and nonsecretory pathways. Cancer Res..

[21] Sauane M., Su Z., Dash R., Liu X., Norris J.S., Sarkar D., Lee S., Allegood J.C., Dent P., Spiegel S. (2009). Ceramide plays a prominent role in MDA-7/IL-24-induced cancer-specific apoptosis. J. Cell. Physiol..

[22] Sauane M., Su Z., Gupta P., Lebedeva I., Dent P., Sarkar D., Fisher P.B. (2008). Autocrine regulation of mda-7/IL-24 mediates cancer-specific apoptosis. Proc. Natl. Acad. Sci. USA.

[23] Sarkar D., Su Z., Lebedeva I., Sauane M., Gopalkrishnan R.V., Valerie K., Dent P., Fisher P.B. (2002). *mda-*7 (IL-24) mediates selective apoptosis in human melanoma cells by inducing the coordinated overexpression of the GADD family of genes by means of p38 MAPK. Proc. Natl. Acad. Sci. USA.

[24] Otkjaer K., Holtmann H., Kragstrup T.W., Paludan S.R., Johansen C., Gaestel M., Kragballe K., Iversen L. (2010). The p38 MAPK Regulates IL-24 Expression by Stabilization of the 3′ UTR of IL-24 mRNA. PLoS ONE.

[25] Sahoo A., Yun M.J., Kwon H.K., Yi H.J., Lee S., Chang S., Park Z.Y., Hwang K.C., Im S.H. (2008). A novel splicing variant of mouse interleukin (IL)-24 antagonizes IL-24-induced apoptosis. J. Biol. Chem..

[26] Whitaker E.L., Filippov V., Filippova M., Guerrero-Juarez C.F., Duerksen-Hughes P.J. (2011). Splice variants of mda-7/IL-24 differentially affect survival and induce apoptosis in U2OS cells. Cytokine.

[27] Filippov V., Schmidt E.L., Filippova M., Duerksen-Hughes P.J. (2008). Splicing and splice factor SRp55 participate in the response to DNA damage by changing isoform ratios of target genes. Gene.

[28] Caudell E.G., Mumm J.B., Poindexter N., Ekmekcioglu S., Mhashilkar A.M., Yang X.H., Retter M.W., Hill P., Chada S., Grimm E.A. (2002). The protein product of the tumor suppressor gene, melanoma differentiation-associated gene 7, exhibits immunostimulatory activity and is designated IL-24. J. Immunol..

[29] Ramesh R., Mhashilkar A.M., Tanaka F., Saito Y., Branch C.D., Sieger K., Mumm J.B., Stewart A.L., Boquio A., Dumoutier L. (2003). Melanoma differentiation-associated gene 7/interleukin (IL)-24 is a novel ligand that regulates angiogenesis via the IL-22 receptor. Cancer Res..

[30] Oncology Drugs in the Pipeline. HemOnc Today, 2015. http://m1.wyanokecdn.com/154294cceb75a0f83401b139bc08edf7.pdf.

[31] Chiocca E.A., Rabkin S.D. (2012). Oncolytic viruses and their application to cancer immunotherapy oncolytic viruses. Cancer Immunol. Res..

[32] Zhao L., Gu J., Dong A., Zhang Y., Zhong L., He L., Wang Y., Zhang J., Zhang Z., Huiwang J. (2005). Potent antitumor activity of oncolytic adenovirus expressing mda-7/IL-24 for colorectal cancer. Hum. Gene Ther..

[33] Zhao L., Dong A., Gu J., Liu Z., Zhang Y., Zhang W., Wang Y., He L., Qian C., Qian Q. (2006). The antitumor activity of TRAIL and IL-24 with replicating oncolytic adenovirus in colorectal cancer. Cancer Gene Ther..

[34] Luo J., Xia Q., Zhang R., Lv C., Zhang W., Wang Y., Cui Q., Liu L., Cai R., Qian C. (2008). Treatment of cancer with a novel dual-targeted conditionally replicative adenovirus armed with mda-7/IL-24 gene. Clin. Cancer Res..

[35] Zhang K.J., Zhang J., Wu Y.M., Qiang J., Liu X.J., Yan L.C., Zhou X.M., Xiao R.J., Wang Y.G., Cao X. (2012). Complete eradication of hepatomas using an oncolytic adenovirus containing AFP promoter controlling E1A and an E1B deletion to drive IL-24 expression. Cancer Gene Ther..

[36] Jiang G., Jiang A.J., Cheng Q., Tian H., Li L.T., Zheng J.N. (2013). A dual-regulated oncolytic adenovirus expressing interleukin-24 sensitizes melanoma cells to temozolomide via the induction of apoptosis. Tumor Biol..

[37] Zhang K.J., Wang Y.G., Cao X., Zhong S.Y., Wei R.C., Wu Y.M., Yue X.T., Li G.C., Liu X.Y. (2009). Potent antitumor effect of interleukin-24 gene in the survivin promoter and retinoblastoma double-regulated oncolytic adenovirus. Hum. Gene Ther..

[38] He M., Liang P. (2010). IL-24 transgenic mice: *In vivo* evidence of overlapping functions for IL-20, IL-22, and IL-24 in the epidermis. J. Immunol..

[39] Wu B., Huang C., Kato-Maeda M., Hopewell P.C., Daley C.L., Krensky A.M., Clayberger C. (2007). Messenger RNA expression of IL-8, FOXP3, and IL-12 differentiates latent tuberculosis infection from disease. J. Immunol..

[40] Xiao L., Li X., Niu N., Qian J., Xie G., Wang Y. (2010). Dichloroacetate (DCA) enhances tumor cell death in combination with oncolytic adenovirus armed with MDA-7/IL-24. Mol. Cell. Biochem..

[41] Liu Z., Xu L., Yuan H., Zhang Y., Zhang X., Zhao D. (2015). Oncolytic adenovirus-mediated mda-7/IL-24 expression suppresses osteosarcoma growth and enhances sensitivity to doxorubicin. Mol. Med. Rep..

[42] Lou W., Chen Q., Ma L., Liu J., Yang Z., Shen J., Cui Y., Bian X.W., Qian C. (2013). Oncolytic adenovirus co-expressing miRNA-34a and IL-24 induces superior antitumor activity in experimental tumor model. J. Mol. Med..

[43] Chada S., Sutton R.B., Ekmekcioglu S., Ellerhorst J., Mumm J.B., Leitner W.W., Yang H.Y., Sahin A.A., Hunt K.K., Fuson K.L. (2004). MDA-7/IL-24 is a unique cytokine-tumor suppressor in the IL-10 Family. Int. Immunopharmacol..

[44] Gopalan B., Litvak A., Sharma S., Mhashilkar A.M., Chada S. (2005). Activation of the Fas-FasL signaling pathway by MDA-7/IL-24 kills human ovarian cancer cells. Cancer Res..

[45] Das D., Persaud L., Dejoie J., Happy M., Brannigan O., de Jesus D., Sauane M. (2016). Tumor necrosis factor-related apoptosis-inducing ligand (TRAIL) activates caspases in human prostate cancer cells through sigma 1 receptor. Biochem. Biophys. Res. Commun..

[46] Ramesh R., Ito I., Saito Y., Mhashilkar A., Branch C.D., Chada S. (2004). Overexpression of the melanoma differentiation associated-7 (mda-7)/interleukin-24 (IL-24) gene impairs lung cancer cell migration by modulating matrix metalloproteinase-2 (MMP-2) and E-cadherin expression. Mol. Ther..

[47] Chada S., Bocangel D., Ramesh R., Grimm E.A., Mumm J.B., Mhashilkar A.M., Zheng M. (2005). mda-7/IL24 kills pancreatic cancer cells by inhibition of the Wnt/PI3K signaling pathways: Identification of IL-20 receptor-mediated bystander activity against pancreatic cancer. Mol. Ther..

[48] Akashi T., Koizumi K., Tsuneyama K., Saiki I., Takano Y., Fuse H. (2008). Chemokine receptor CXCR4 expression and prognosis in patients with metastatic prostate cancer. Cancer Sci..

[49] Panneerselvam J., Munshi A., Ramesh R. (2013). Molecular targets and signaling pathways regulated by interleukin (IL)-24 in mediating its antitumor activities. J. Mol. Signal..

[50] Panneerselvam J., Jin J., Shanker M., Lauderdale J., Bates J., Wang Q., Zhao Y.D., Archibald S.J., Hubin T.J., Ramesh R. (2015). IL-24 Inhibits lung cancer cell migration and invasion by disrupting the SDF-1/CXCR4 signaling axis. PLoS ONE.

[51] Bech R., Otkjaer K., Birkelund S., Vorup-Jensen T., Agger R., Johansen C., Iversen L., Kragballe K., Rømer J. (2014). Interleukin 20 protein locates to distinct mononuclear cells in psoriatic skin. Exp. Dermatol..

[52] Kumari S., Bonnet M.C., Ulvmar M.H., Wolk K., Karagianni N., Witte E., Uthoff-Hachenberg C., Renauld J.C., Kollias G., Toftgard R. (2013). Tumor necrosis factor receptor signaling in keratinocytes triggers interleukin-24-dependent psoriasis-like skin inflammation in mice. Immunity.

[53] Sa S.M., Valdez P.A., Wu J., Jung K., Zhong F., Hall L., Kasman I., Winer J., Modrusan Z., Danilenko D.M. (2007). The effects of IL-20 subfamily cytokines on reconstituted human epidermis suggest potential roles in cutaneous innate defense and pathogenic adaptive immunity in psoriasis. J. Immunol..

[54] Kragstrup T.W., Otkjaer K., Holm C., Jørgensen A., Hokland M., Iversen L., Deleuran B. (2008). The expression of IL-20 and IL-24 and their shared receptors are increased in rheumatoid arthritis and spondyloarthropathy. Cytokine.

[55] Fonseca-Camarillo G., Furuzawa-Carballeda J., Granados J., Yamamoto-Furusho J.K. (2014). Expression of interleukin (IL)-19 and IL-24 in inflammatory bowel disease patients: A cross-sectional study. Clin. Exp. Immunol..

[56] Tamai H., Miyake K., Yamaguchi H., Takatori M., Dan K., Inokuchi K., Shimada T. (2012). AAV8 vector expressing IL24 efficiently suppresses tumor growth mediated by specific mechanisms in MLL/AF4-positive ALL model mice. Blood.

[57] Jin S.H., Choi D., Chun Y.J., Noh M. (2014). Keratinocyte-derived IL-24 plays a role in the positive feedback regulation of epidermal inflammation in response to environmental and endogenous toxic stressors. Toxicol. Appl. Pharmacol..

[58] Ma Y., Chen H., Wang Q., Luo F., Yan J., Zhang X.L. (2009). IL-24 protects against Salmonella typhimurium infection by stimulating early neutrophil Th1 cytokine production, which in turn activates CD8+ T cells. Eur. J. Immunol..

[59] Bastonero S., Le Priol Y., Armand M., Bernard C.S., Reynaud-Gaubert M., Olive D., Parzy D., de Bentzmann S., Capo C., Mege J.-L. (2009). New microbicidal functions of tracheal glands: Defective anti-infectious response to Pseudomonas aeruginosa in cystic fibrosis. PLoS ONE.

[60] Wu B., Huang C., Kato-Maeda M., Hopewell P.C., Daley C.L., Krensky A.M., Clayberger C. (2008). IL-24 modulates IFN-γ expression in patients with tuberculosis. Immunol. Lett..

[61] Buzas K., Megyeri K. (2006). Staphylococci induce the production of melanoma differentiation-associated protein-7/IL-24. Acta Microbiol. Immunol. Hung..

[62] Miller L.S., Cho J.S. (2011). Immunity against *Staphylococcus aureus* cutaneous infections. Nat. Rev. Immunol..

[63] Cho J.S., Pietras E.M., Garcia N.C., Ramos R.I., Farzam D.M., Monroe H.R., Magorien J.E., Blauvelt A., Kolls J.K., Cheung A.L. (2010). IL-17 is essential for host defense against cutaneous *Staphylococcus aureus* infection in mice. J. Clin. Investig..

[64] Myles I.A., Fontecilla N.M., Valdez P.A., Vithayathil P.J., Naik S., Belkaid Y., Ouyang W., Datta S.K. (2013). Signaling via the IL-20 receptor inhibits cutaneous production of IL-1β and IL-17A to promote infection with methicillin-resistant *Staphylococcus aureus*. Nat. Immunol..

[65] Bosanquet D.C., Harding K.G., Ruge F., Sanders A.J., Jiang W.G. (2012). Expression of IL-24 and IL-24 receptors in human wound tissues and the biological implications of IL-24 on keratinocytes. Wound Repair Regen..

[66] Poindexter N.J., Williams R.R., Powis G., Jen E., Caudle A.S., Chada S., Grimm E.A. (2010). IL-24 is expressed during wound repair and inhibits TGFα-induced migration and proliferation of keratinocytes. Exp. Dermatol..

[67] Tan Y., Sanders A.J., Zhang Y., Martin T.A., Owen S., Ruge F., Jiang W.G. (2015). Interleukin-24 (IL-24) expression and biological impact on HECV endothelial cells. Cancer Genom. Proteom..

[68] Lee K.M., Kang H.A., Park M., Lee H.Y., Choi H.R., Yun C.H., Oh J.W., Kang H.S. (2012). Interleukin-24 attenuates β-glycerophosphate-induced calcification of vascular smooth muscle cells by inhibiting apoptosis, the expression of calcification and osteoblastic markers, and the Wnt/β-catenin pathway. Biochem. Biophys. Res. Commun..

[69] Chen J., Chada S., Mhashilkar A., Miano J.M. (2003). Tumor suppressor MDA-7/IL-24 selectively inhibits vascular smooth muscle cell growth and migration. Mol. Ther..

[70] Lee K.M., Kang H.A., Ko C.B., Oh E.H., Park M., Lee H.Y., Choi H.R., Yun C.H., Jung W.W., Oh J.W. (2013). Differential gene expression profiles in spontaneously hypertensive rats induced by administration of enalapril and nifedipine. Int. J. Mol. Med..

[71] Vargas-Alarcón G., Posadas-Romero C., Villarreal-Molina T., Alvarez-León E., Angeles-Martinez J., Posadas-Sanchez R., Monroy-Muñoz I., Luna-Fuentes S., González-Salazar C., Ramirez-Bello J. (2014). IL-24 gene polymorphisms are associated with cardiometabolic parameters and cardiovascular risk factors but not with premature coronary artery disease: The genetics of atherosclerotic disease Mexican study. J. Interferon Cytokine Res..

[72] Gupta P., Walter M.R., Su Z.Z., Lebedeva I.V., Emdad L., Randolph A., Valerie K., Sarkar D., Fisher P.B. (2006). BiP/GRP78 is an intracellular target for MDA-7/IL-24 induction of cancer-specific apoptosis. Cancer Res..

[73] Pataer A., Vorburger S.A., Chada S., Balachandran S., Barber G.N., Roth J.A., Hunt K.K., Swisher S.G. (2005). Melanoma differentiation-associated gene-7 protein physically associates with the double-stranded RNA-activated protein kinase PKR. Mol. Ther..

[74] Cruz A., Nguyen B., Sauane M., Lopez G.E. (2016). Structural and functional characterization of interleukin-24 based on atomistic molecular modeling. Chem. Lett..

